# Allogeneic hematopoietic stem cell transplantation after azacitidine and venetoclax salvage in relapsed/refractory AML: a multicenter real-world study by the French AURAML group

**DOI:** 10.1038/s41409-026-02834-z

**Published:** 2026-03-24

**Authors:** Urbain Tauveron-Jalenques, Gaspar Aspas Requena, Zofia Gross, Emmanuelle Tavernier, Pedro Chorão, Martin Carre, Jérôme Cornillon, Adrien Contejean, Clémence Santana, Clément Rocher, Sylvain Lamure, Natacha Mauz, Ugo Thevenet, Nadine Boullanger, Gian Matteo Pica, Arthur Dony, Mauricette Michallet, Amine Belhabri, Maël Heiblig

**Affiliations:** 1 Service d’hématologie et de thérapie cellulaire, CHU Clermont-Ferrand, Clermont-Ferrand, France; 2 Service d’hématologie, CHU Lyon Sud, Lyon, France; 3 Service d’hématologie clinique et thérapie cellulaire, CHU Saint Etienne, Saint Etienne, France; 4 Servicio de Hematología y Hemoterapia, Hospital Universitario y Politécnico La Fe, Valencia, Spain; 5 Service d’hématologie, CHU Grenoble Alpes, La Tronche, France; 6 Service d’hématologie, CH Annecy Genevois, Epagny Metz-Tessy, France; 7 Service d’hématologie, CH de Valence, Valence, France; 8 Service d’hématologie, Centre Léon Bérard, Lyon, France; 9 Service d’hématologie, CH Nord Dauphiné, Bourgoin-Jallieu, France; 10 Service d’hématologie, Hôpitaux Nord-Ouest, Villefranche-sur-Saône, France; 11 Service d’hématologie, CH de Roanne, Roanne, France; 12 Service d’hématologie, CH Métropole Savoie, Chambéry, France

**Keywords:** Acute myeloid leukaemia, Stem-cell therapies

## Abstract

Fit relapsed/refractory (R/R) acute myeloid leukemia (AML) patients usually undergo intensive chemotherapy (IC)-based salvage to bridge them to allogeneic hematopoietic stem cell transplantation (HSCT), but their prognosis remains poor. Azacitidine and venetoclax (AZA/VEN) are increasingly used as salvage therapy in R/R AML with encouraging results, although data remain limited. In this study, we evaluated the post-HSCT outcomes of 75 R/R AML patients from the VENAURA registry who underwent HSCT after AZA/VEN salvage. After a median follow-up of 16.9 months, the estimated 2-year overall survival (OS) was 61.4% (95% confidence interval [CI]: 49.5–68.1%). The 2-year cumulative incidence of relapse (CIR) was 35.1% (95% CI: 20–50.2%). The estimated 2-year non-relapse mortality (NRM) rate was 10.6% (95% CI: 9.8–23.3%). Cytological response at the end of cycle 1 was independently associated with OS and CIR in multivariate analysis. Comparison with 75 pair-matched patients receiving IC-based salvage prior to HSCT revealed similar OS in both groups. CIR was not significantly higher in AZA/VEN-treated compared to IC-treated patients; however, there was a trend toward a lower 2-year NRM rate in the AZA/VEN group. Our data suggest that AZA/VEN represents a feasible bridge-to-transplant option with a favorable toxicity profile.

## Introduction

Despite continual advances in treatment, acute myeloid leukemia (AML) remains a high-risk malignancy with approximately 20% of patients presenting primary refractory disease after conventional intensive chemotherapy (IC) and more than 50% of patients attaining a first complete remission (CR1) after IC eventually relapsing [1, 2]. As a result, the long-term prognosis of relapsed/refractory (R/R) AML is poor, with a 5-year overall survival (OS) of only ~10% [3]. In the absence of an established standard of care, fit patients with refractory or relapsed (R/R) disease are usually treated with a variety of salvage therapies in order to bridge them to allogenic hematopoietic stem cell transplantation (HSCT), the only potentially curative treatment in this setting. However, IC-based salvage strategies yield heterogeneous response rates and are frequently limited by significant toxicity [1–3].

Following the results of the randomized phase 3 VIALE-A clinical trial, the association of azacitidine (AZA) and the B-cell lymphoma 2 (BCL2) inhibitor venetoclax (VEN) became the standard of care for newly diagnosed (ND) AML in patients ineligible to conventional IC [4]. Although initially developed for this unfit population, AZA/VEN has been increasingly used off-label in the relapsed/refractory (R/R) setting, including in fit, transplant-eligible patients [5–12]. In addition, several retrospective studies have reported encouraging post-transplant outcomes in patients undergoing allo-HSCT after achieving first remission with AZA/VEN, with favorable overall survival, relapse rates, and transplant-related mortality [13–16].

However, data regarding the efficacy and safety of HSCT after AZA/VEN salvage remains limited [17–20]. Notably, in the series published by Unglaub et al., VEN-based non-intensive salvage led to a 62% complete remission (CR)/CR with incomplete hematological recovery (CRi) rate. Seventy-three percent of patients subsequently underwent HSCT, and the median OS of VEN-treated patients was 15.8 months [19]. In this multicenter retrospective study, we aimed to evaluate the post-transplant outcomes of R/R AML patients from the VENAURA registry who underwent HSCT after AZA/VEN salvage.

## Material and methods

### Study design

VENAURA is a retrospective registry including 592 AZA/VEN-treated AML patients from 12 centers in the French Auvergne-Rhône-Alpes (AURA) region from January 2019 to February 2024 (IRB00013204). Adult patients with R/R AML treated in frontline settings with IC or hypomethylating agent (HMA) monotherapy for pre-existing high-risk myelodysplastic syndrome (MDS) were included. At diagnosis, peripheral blood (PB) and bone marrow (BM) samples were examined for cytogenetic abnormalities and molecular markers according to local procedures. Patients were stratified according to the European LeukemiaNet (ELN) 2022 and 2024 risk classifications [21] while adverse cytogenetic abnormalities were defined according to the ELN 2022 classification [1]. Thirteen types of conditioning regimen were used, with a majority of reduced intensity conditioning (RIC) (detailed in Supplementary Table 1) [22]. Patients received in vivo T-cell depletion using anti-thymocyte globulin (ATG) 5 mg/kg total dose for HLA-matched donors (matched sibling and matched unrelated donors) and post-transplant cyclophosphamide for HLA-mismatched donors (haplo-identical and mismatched unrelated donors). graft-versus-host disease (GVHD) prophylaxis was mainly based on cyclosporine alone, mycophenolate mofetil and cyclosporine, or methotrexate/cyclosporine in cases of transplant with minor ABO incompatibility.

All patients were included in the European internet-based ProMISe database and gave informed consent for the collection of their personal data in this database. Extraction and analysis of data were performed in accordance with local ethics committee requirements (CNIL2093819).

### AZA/VEN response and MRD assessment

CR and refractory disease were defined according to international recommendations [1]. Composite CR (CRc) was defined as patients reaching CR, CRi, or morphologic leukemia-free state (MLFS) at any time. Multiparameter flow cytometry-based minimal residual disease (MFC-MRD) and/or NPM1/WT1 minimal residual disease by RT-PCR (RT-PCR-MRD) were assessed within 4 weeks before transplant. Regarding flow cytometry-based MRD, its assessment was based on leukemia-associated immunophenotype (LAIP) using the ELN recommendations (bulk lyses, and at least 500,000 recorded events to achieve a sensitivity threshold of at least 10^-3^ (0.1%)) [23]. MFC-MRD was performed in BM samples using an 8-color panel and considered positive when detectable up to 0.1% threshold [24–26]. For RT-PCR-MRD, MRD negativity (MRD^neg^) was defined as ≤ 10-4 for NPM1 by RT-qPCR and ≤10-3 for WT1 by RT-qPCR on PB. For NPM1 mutated patients evaluated by both MFC- and RT-PCR-MRD, discordant results (MFC negative but RT-qPCR positive) were classified as MRD^pos^.

### Endpoints–definitions

The primary endpoint was OS. Secondary endpoints included cumulative incidence of relapse (CIR), non-relapse mortality (NRM), refined GVHD and relapse-free survival (GRFS), acute GVHD (aGVHD) and chronic GVHD (cGVHD). Secondary malignancies with fatal issues were considered HSCT-related deaths. aGVHD cases were reported during the first 100 days following HSCT, and severity was graded according to the modified Glücksberg criteria [27]. cGVHD was staged according to the National Institutes of Health consensus criteria [28, 29]. Refined GRFS was defined as survival without grade III to IV aGVHD, severe cGVHD, relapse and deaths from any cause after HSCT and was calculated from the date of HSCT. Patients were considered refractory when the disease was not in CRc at the time of HSCT.

### Pair-matching cohort

In order to compare post-transplant outcomes to those after conventional IC-based salvage, we pair-matched at a 1:1 ratio the VENAURA cohort to a local cohort (Lyon Sud Hospital, France) of R/R patients treated with IC prior to HSCT between August 2006 and December 2021. Variables used for pair-matching were age, sex, pre-HSCT cytologic response, pre-HSCT MRD response, donor type, Hematopoietic cell transplantation-specific comorbidity index (HCT-CI) and conditioning regimen intensity [30]. All patients included in the pair-matching cohort were also included in the European internet-based ProMISe database and gave informed consent for the collection of their personal data in this database. Extraction and analysis of data were performed in accordance with local ethics committee requirements (CNIL2093819).

### Statistical analysis

For comparisons between patients, disease and transplant-related characteristics, Mann-Whitney and Kruskal-Wallis tests for continuous quantitative variables, and Chi-square tests for categorical variables were performed. Probabilities of OS and GRFS were calculated using the Kaplan-Meier method. To study aGVHD, cGVHD, CIR and NRM, a cumulative incidence model was used. Univariate analyses were carried out using log-rank tests for OS and GRFS, and the method of Gray for cumulative incidence outcome variables. Multivariate regression was performed, including all significant variables in univariate analyses (defined as a *p*-value ≤ 0.05), using a Cox proportional hazard model. Statistical analyses were performed using GraphPad Prism software version 8.0.1 for Windows (GraphPad Software, San Diego, California, USA, www.graphpad.com) and R software version 4.1.1. (R Core Team 2021, R: A language and environment for statistical computing, R Foundation for Statistical Computing, Vienna, Austria, https://www.R-project.org).

## Results

### Patients and HSCT characteristics

In the VENAURA registry, 305 patients received AZA/VEN as a salvage therapy in relapsed/refractory settings post-intensive IC (*n* = 240), after HMA monotherapy failure for high-risk MDS (*n* = 29) or for molecular relapse (*n* = 36). In patients who received AZA/VEN for morphological relapse/progression, CRc rate was 61.4 and 51.7% in post-IC and post-HMA settings, respectively. HSCT rate in CRc patients was 34 and 26.6% in post-IC and post-HMA settings, respectively. For patients in molecular relapse, 88.8% (32/36) attained molecular response or stable disease, and 17 out of 32 (53.1%) moved to transplant (Supplementary Fig. 1). Overall, 75 patients received an HSCT after AZA/VEN salvage. With a median age of 58.2 years, the median number of treatment lines prior to AZA/VEN salvage was 1 (range: 1–3). At diagnosis, patients had favorable, intermediate and adverse risk disease according to the ELN 2022 classification in 12.3, 26 and 61.6% and the 2024 classification in 61.3, 16 and 20% of cases, respectively (missing data: 2/75). Adverse cytogenetics according to ELN 2022 were reported in 22/75 (29.3%) of cases, including 18/75 (24%) with complex karyotype. Patients’ characteristics and detailed molecular characteristics are summarized in Table 1 and Supplementary Fig. 2.

**Table 1:** Patients baseline characteristics.

Patients characteristics	*N* = 75
**Age**, years, median (range)	58.2 (18–73)
**Female sex**, *n* (%)	36/75 (48)
**ELN 2022 risk classification**, *n* (%) • Favorable • Intermediate • Adverse	9/73 (12.3) 19/73 (26) 45/73 (61.6)
**ELN 2024 risk classification**, *n* (%) • Favorable • Intermediate • Adverse	46/73 (63) 15/73 (20) 12/73 (17)
**Intensive chemotherapy lines prior to AZA/VEN salvage**, *n* (%) • 1 • 2	58/75 (77.3) 17/75 (22.7)
**AML status at AZA/VEN onset**, *n* (%) • Refractory to IC • Relapse after IC • Molecular relapse after IC	35/75 (46.7) 32/75 (42.6) 8/75 (10.7)
**Number of AZA/VEN cycles pre-HSCT**, median (range)	2 (1–6)
**Pre-HSCT response**, *n* (%) • CR • CRi/MLFS • Refractory	55/75 (73.3) 16/75 (21.3) 4/75 (5.4)
**MFC pre-HSCT MRD**, *n* (%)* • Negative • Positive • Not realized	46/71 (64.7) 20/71 (28.1) 5/71 (7)
**HSCT characteristics**	***N*** = **75**
**Donor type**, *n* (%) • MSD • MUD • MMUD • Haplo-identical	12/75 (16) 38/75 (50.6) 5/75 (6.7) 20/75 (26.7)
**HCT-CI**, *n* (%) • 0 • 1 • 2 • ≥ 3	41/75 (54.6) 16/75 (21.3) 11/75 (14.7) 7/75 (9.4)
**Conditioning regimens**, *n* (%) • RIC • MAC • Sequential	53/75 (70.7) 9/75 (12) 13/75 (17.3)
**Post transplantation GVHD prophylaxis**, *n* (%) • CSA • CSA + MMF • CSA + MTX	21/75 (28) 42/75 (56) 12/75 (16)

* Excluding 4 patients with refractory disease at the time of HSCT.

*AML* acute myeloid leukemia, *AZA/VEN* azacitidine and venetoclax, *CR* complete response, *CRi* Complete response with incomplete hematological recovery, *CSA* cyclosporine, *ELN* European LeukemiaNet, *GVHD* graft-versus-host disease, *HCT-CI* hematopoietic cell transplantation-specific comorbidity index, *HSCT* allogenic hematopoietic stem cell transplantation, *MAC* myeloablative conditioning, *MFC* multiparameter flow cytometry, *MMF* mycophenolate mofetil, *MLFS* morphologic leukemia-free state, *MMUD* mismatched unrelated donor, *MRD* minimal residual disease, *MSD* matched sibling donor, *MTX* methotrexate, *MUD* matched unrelated donor, *RIC* reduced intensity conditioning.

Regarding AZA/VEN treatment prior to HSCT, patients received a median of 2 cycles (range: 1–6). Overall, 55/75 (73.3%) achieved CRc at the end of cycle 1, while 18/75 (24%) responded in later cycles. Four patients were refractory to AZA/VEN at the time of HSCT. Median time from CRc to HSCT was 2.14 months (95% CI: 1.04–3.30). Pre-HSCT MRD by MFC (*n* = 54) and/or WT1/NPM1 RT-qPCR (*n* = 12) was available in 66/71 (92.9%) responding patients. Overall, 46 (64.7%) and 20 (28.1%) were MRD^neg^ and MRD^pos,^ respectively.

HSCT characteristics are summarized in Table 1. Most patients received reduced intensity conditioning (RIC) regimens (70.3%). Stem cell sources were peripheral blood, bone marrow, and cord blood in 94.8%, 2.6%, and 2.6% of patients, respectively. Donor/recipient (D/R) CMV status prior to HSCT was D–/R–, D–/R + , D + /R– and D + /R+ in 28.0%, 29.3%, 9.3% and 33.4% of cases, respectively. Conditioning regimens are detailed in Supplementary Table 1.

### Engraftment

At day +30, 66/71 evaluable patients (92.9%) had recipient marrow chimerism <5%, whereas 5 patients (7%) exhibited ≥5% recipient chimerism. Only 2 of them relapsed during follow-up. At day +90, 61/75 patients were evaluable for donor marrow chimerism. Partial chimerism (defined at day+90 as recipient chimerism ≥1%) was observed in 14/61 (22.9%) of patients.

### Overall outcome and impact of pre-HSCT response to AZA/VEN

**Fig. 1:**
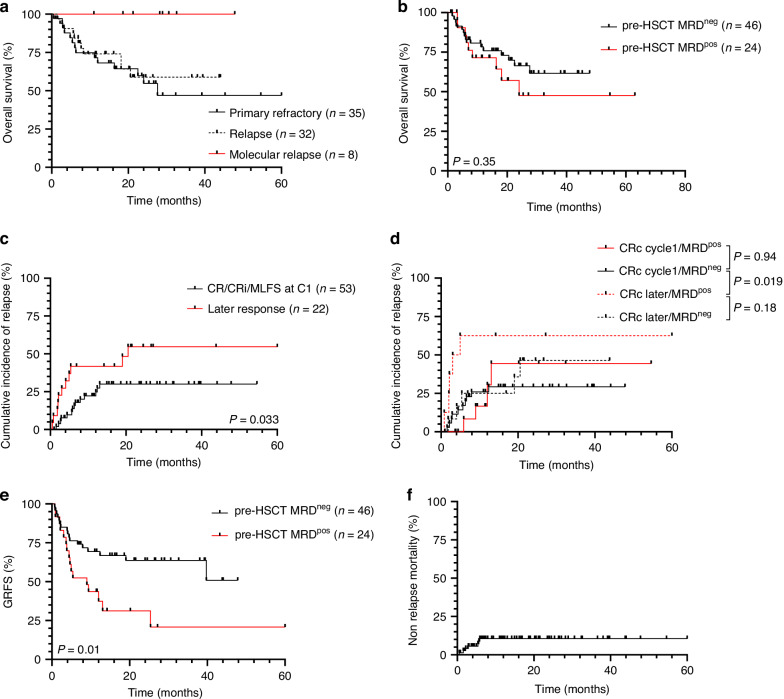
Post-HSCT outcomes analysis for subgroups of AZA/VEN-treated patients. Kaplan-Meier estimates of OS according to disease status prior to AZA/VEN salvage (**a**) and pre-HSCT MRD status (**b**); CIR according to timing of AZA/VEN response (**c**) and MRD at the time of HSCT (**d**); GRFS according to pre-HSCT MRD status (**e**) and NRM (**f**).

After a median follow-up of 16.9 months (range: 0.53–63.3), median OS was not reached and the estimated 2-year OS was 61.4% (95% CI: 49.5–68.1%) (Supplementary Fig. 3). Post IC refractory or relapsed status prior to AZA/VEN did not significantly influence post-transplant OS. However, patients in molecular relapse salvaged by AZA/VEN and bridged to HSCT had a post-transplant OS of 100% (Fig. 1a). The number of IC lines prior to AZA/VEN salvage had no impact on post-HSCT survival. While ELN 2022 and 2024 risk classification at diagnosis did not impact OS, adverse cytogenetics according to ELN 2022 were associated with a poor outcome (median OS: 18.1 months) compared to diploid karyotypes (median OS NR; HR = 2.52, *p* = 0.028) (Supplementary Table 2). Type of response (CR/CRi vs MLFS) was associated with a similar probability of 24 months OS (64.2 vs 58.4% respectively, *p* = 0.56). Regarding relapse risk, the 2-year CIR was 35.1% (95% CI: 20–50.2%) (Supplementary Fig. 4).

Median OS was 24 months in MRD^pos^ patients and not reached (NR) in MRD^neg^ cases, yet this difference was not statistically significant (HR = 1.48; 95% CI: 0.28–1.65; *p* = 0.35). (Fig. 1b). However, absence of response at the end of AZA/VEN cycle 1 was associated with a worse OS compared to patients who responded at the end of cycle 1 (HR = 5.41; 95% CI: 1.97–14.87; *p* < 0.001). Conversely, CIR was significantly higher in patients who did not reach CRc at the end of cycle 1 (late responders) compared to early responders, with a median time for relapse of 20.5 months and NR, respectively (HR = 2.6; 95% CI: 1.1–6.6; *p* = 0.033) (Fig. 1c). Late responders with a pre-HSCT MRD^pos^ (*n* = 8) had a 2-year CIR of 62.5%. Conversely, early responders with a pre-HSCT MRD^neg^ (*n* = 35) had a 2-years CIR of 29.6% (*p* = 0.019). Early responders/pre-HSCT MRD^pos^ and late responders/pre-HSCT MRD^neg^ patients had an intermediate risk of relapse with a 2-year CIR of 44.4 and 46.4%, respectively (Fig. 1d).

Univariate analyses for OS and CIR are reported in Supplementary Table 2 and 3. In the multivariate analysis for OS, response at the end of AZA/VEN cycle 1 was the only variable independently associated with survival (HR = 4.94; *p* = 0.009), while karyotype, ELN 2024 risk group and pre-HSCT MRD were not associated with OS (Table 2). Regarding CIR, ELN 2024 risk group at diagnosis (adverse vs favorable; HR = 0.13, *p* = 0.013), AZA/VEN cycle 1 response (no response *vs* CR/CRi/MLFS; HR = 3.23; *p* = 0.009), but also pre-HSCT MRD (MRD^pos^ vs MRD^neg^; HR = 3.51; *p* = 0.042) were independent variables associated with relapse risk (Table 2).

**Table 2:** Multivariate analysis of post-HSCT outcomes in AZA/VEN-treated patients.

Characteristics		HR	95% CI	*P*-value
Overall survival				
AML type	MR vs de novo	3.57	0.86–14.8	0.08
	sAML vs de novo	2.46	0.7–8.7	0.16
Karyotype	Miscellaneous vs diploid	0.19	0.02–1.91	0.16
	Adverse vs diploid	1.38	0.38–5.05	0.62
ELN 2024	Favorable vs adverse	0.85	0.17–4.35	0.85
	Intermediate vs adverse	2.04	0.28–14.6	0.47
C1 response	No response vs CR/CRi/MLFS	4.94	1.93–14.3	0.009
**Cumulative incidence of relapse (CIR)**				
Sex	Male vs female	3.9	1.33–11.4	0.13
Karyotype	Miscellaneous vs diploid	0.27	0.3–2.32	0.23
	Adverse vs diploid	0.86	0.2–3.61	0.83
ELN 2024	Favorable vs adverse	0.13	0.02–0.98	0.013
	Intermediate vs adverse	0.54	0.07–4.3	0.55
C1 response	No response vs CR/CRi/MLFS	3.23	1.93–14.9	0.009
Pre-HSCT MRD response	MRD^pos^ vs MRD^neg^	3.51	1.91–12	0.042
**Graft-versus-host disease-free, relapse-free survival (GRFS)**				
AML type at diagnosis	MR vs de novo	2.47	0.74–8.26	0.14
	sAML vs de novo	0.79	0.23–2.72	0.70
Karyotype	Miscellaneous vs diploid	0.15	0.02–1.23	0.08
	Adverse vs diploid	0.95	0.28–3.17	0.93
ELN 2024 risk classification	Favorable vs adverse	0.53	0.13–2.16	0.85
	Intermediate vs adverse	0.51	0.09–2.96	0.47
C1 response	No response vs CR/CRi/MLFS	5	1.64–15.2	0.005
Pre-HSCT MRD response	MRD^pos^ vs MRD^neg^	1.98	0.76–5.1	0.16

*AML* acute myeloid leukemia, *C1* cycle 1, *CR* complete response, *CRi* complete response with incomplete hematological recovery, *ELN* European Leukemia Net, *MLFS* marrow leukemia free state, *MR* myelodysplasia-related, *MRD* measurable residual disease, *sAML* secondary AML.

### GVHD, GRFS and NRM

Acute GVHD occurred after a median time of 53 days (range: 26–118). The cumulative incidence of grade 2–4 and 3–4 aGVHD at day+100 were 12% (95% CI: 6.1–18.8%) and 6.6% (95% CI: 1.4–9.3%), respectively. No predictive factors for aGVHD were identified. cGVHD occurred after a median time of 6 months (range: 2.4–16.6). Cumulative incidence of all grades cGVHD and extensive cGVHD at 2 years were 22.6% (95% CI: 15.9–33.7%) and 5.3% (95% CI: 2.8–10%), respectively. Only donor CMV status (positive vs negative, HR = 3.71; 95% CI: 1.24–12; *p* = 0.02) was associated with a higher risk of developing cGVHD, while GVHD prophylaxis type, conditioning regimen or type of donor were not. Overall, median GRFS was 39.7 months with a 2-year estimated GRFS of 53.1% (95% CI: 30.7–54.1%) (Supplementary Fig. 5). Patients with a pre-HSCT MRD^pos^ had a median GRFS of 8.3 months, whereas median GRFS was NR for those with a pre-HSCT MRD^neg^ response (Fig. 1e). Univariate analysis for GRFS is detailed in Supplementary Table 4. In multivariate analysis, only AZA/VEN cycle 1 response (no response *vs* CR/CRi/MLFS; HR = 5; 95% CI: 1.64–15.2; *p* = 0.005) was independently associated with GRFS, while pre-HSCT MRD (MRD^pos^ vs MRD^neg^) was not. The 2-year NRM was low at 10.6% (95% CI: 9.8–23.3%) (Fig. 1f and Table 2). History of prior myeloid neoplasm or therapy-related AML and myelodysplasia-related AML diagnosis tended to be associated with NRM (Supplementary Table 5). Among the 8 transplant-related deaths, 3 were attributable to infections and 5 to refractory GVHD.

### Pair matching with patients treated with intensive chemotherapy as a salvage pre-HSCT

**Fig. 2:**
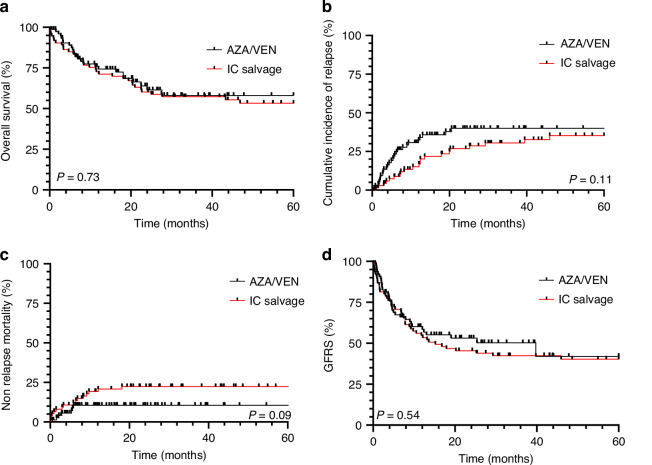
Post-HSCT outcomes analysis for AZA/VEN and IC-treated patients. Kaplan-Meier estimates of OS (**a**), CIR (**b**), NRM (**c**) and GRFS (**d**) of pair-matched AZA/VEN and IC salvaged cohorts.

In order to compare AZA/VEN to IC as a salvage strategy in R/R AML, we used a retrospective local cohort of patients (*n* = 75) salvaged by conventional IC (IC cohort) pair matched to the AZA/VEN cohort on potential confounding factors influencing post-HSCT outcomes (age, cytogenetics, number of treatment lines prior to HSCT, cytologic response at the time of HSCT, pre-HSCT MRD status, donor type, conditioning regimen). Regarding the type of pre-HSCT salvage in the IC cohort, intermediate-dose cytarabine-, anthracyclines- and GO-based salvages were used in 13.3%, 57.3%, and 29.4% of cases, respectively. Overall, both cohorts were comparable (Supplementary Table 6). There was no significant difference regarding OS whether patients received AZA/VEN or IC pre-HSCT (Fig. 2a). While CIR was numerically higher in AZA/VEN treated patients (2-years CIR: 35.1%) compared to IC salvaged patients (2-years CIR: 26.6%), this difference was not statistically significant (*p* = 0.11) (Fig. 2b). Conversely, there was a trend toward a lower 2-years NRM rate in AZA/VEN treated patient (10.6%) compared to IC salvaged patients (25.1%, *p* = 0.09) (Fig. 2c). As for OS, there was no difference between AZA/VEN and IC salvaged patients regarding GRFS (Fig. 2d).

## Discussion

Management of R/R AML remains challenging, with HSCT representing the only potentially curative option for eligible patients in this setting. IC salvage has long been the standard to induce remission before transplant. However, IC response rates are limited and associated with significant treatment-related toxicities that can compromise subsequent eligibility to transplant [1–3]. This underscores the unmet need for effective bridge-to-transplant strategies that achieve disease control with reduced toxicity. In this context, VEN has changed the therapeutic landscape of AML, initially by improving outcomes in patients unfit for IC, and more recently through accumulating evidence supporting the efficacy of VEN-based combinations in the R/R setting [5–12]. Dumas et al. recently reported that AZA/VEN salvage in primary refractory or relapsed after IC AML patients was associated with similar response rates compared to intermediate dose cytarabine-based salvage [31]. Despite these encouraging results, data on the feasibility and outcomes of HSCT following AZA/VEN salvage in R/R AML remain scarce, particularly in real-world settings. Most available studies are limited by small patient numbers or heterogeneous populations. In this context, our multicenter retrospective study aimed to address this gap by evaluating the post-transplant outcomes of patients treated with AZA/VEN salvage within the VENAURA registry and by directly comparing them, through a pair-matched analysis, to a cohort of patients receiving conventional IC prior to HSCT.

Notwithstanding the retrospective design, our study represents a large multicenter cohort of patients with R/R AML treated with AZA/VEN as salvage prior to allo-HSCT. In our cohort, AZA/VEN salvage treatment yielded an HSCT rate of 26-36% in morphological relapse/refractory patients that reached CRc after AZA/VEN. However, this result has to be taken with caution as the VENAURA retrospective registry did not capture the pre-AZA/VEN intention to transplant status, patient or donor availability at AZA/VEN onset. Altogether, this result may not reflect the full potential of AZA/VEN accurately as a bridge to transplant. Indeed, the Study Alliance Leukemia reported an HSCT rate of 73% in AZA/VEN-responding patients with an intention to transplant in a large retrospective study [19].

Nevertheless, our results show that this strategy is both effective and safe, with a 2-year OS exceeding 60%, which compares favorably with historical series of patients receiving IC in older populations [32]. Particularly, patients rescued at the stage of molecular relapse with AZA/VEN achieved excellent outcomes after HSCT, with survival rates markedly superior to those usually observed in overt hematologic relapse, in line with recently published studies that confirm our findings [33]. Moreover, 2-year cumulative NRM was remarkably low (10.6%), substantially lower than the rates typically reported after IC-based salvage, and consistent with the hypothesis that AZA/VEN allows disease control while minimizing additional treatment-related toxicity before transplantation. We can hypothesize that, by reducing tissular exposure to highly cytotoxic agents prior to HSCT, AZA/VEN may limit pre-transplant inflammatory burden but also help preserve patient fitness, thereby reducing high-grade GVHD and post-transplant complications. This observation was further reinforced by the pair-matched analysis, which demonstrated comparable post-transplant survival between patients salvaged with AZA/VEN or IC, while NRM tended to be lower with AZA/VEN. Taken together, these results support the use of AZA/VEN as a feasible bridge-to-transplant approach in the real-world setting of R/R AML. Its favorable safety profile makes this approach particularly appealing for older patients and those with relevant comorbidities, while its efficacy appears comparable to that achieved with conventional intensive induction or reinduction chemotherapy.

In our study, the most discriminant factor for survival was an early response (CRc by the end of cycle 1) to AZA/VEN. While early compared to late responders do not seem to have different outcomes in frontline settings [4], optimizing AZA/VEN cycle 1 course in order to reach early CRc is critical when used as a bridge to HSCT. Besides cytologic response, the predictive value on outcome of pre-HSCT MRD response in AZA/VEN salvaged patients is unknown. Numerous studies have shown that MRD status at the time of HSCT in patients salvaged with IC was significantly associated with post-transplant outcomes [34–37].

While pre-HSCT MRD assessment also showed prognostic value, particularly when integrated with response kinetics, it did not retain significance as an independent factor. Based on these observations, we propose a simple pre-transplant stratification model with three groups: patients achieving an early response and MRD^neg^ with favorable outcomes, those with late response and persistent MRD^pos^ with poor prognosis, and an intermediate group defined by early responders remaining MRD^pos^ or late responders achieving MRD^neg^. This model needs further validation on large independent cohorts.

With regard to CIR, our findings highlight that response kinetics are as critical as MRD status. Patients achieving late responses were at higher risk of post-transplant relapse, particularly when MRD^pos^ persisted, whereas early responders who achieved MRD^neg^ showed excellent disease control, with a 2-year CIR close to 30%. These observations suggest that combining early response assessment with MRD evaluation may refine pre-transplant risk stratification and better identify patients who could benefit from additional pre- or post-transplant strategies.

The safety profile of AZA/VEN as a bridge to HSCT was also favorable in our cohort. The cumulative incidence of severe aGVHD (grade III–IV) remained below 10%, and extensive cGVHD was uncommon. These findings are consistent with the hypothesis that AZA/VEN does not add significant organ toxicity prior to transplant and may thus help to preserve patient fitness at the time of conditioning. Importantly, our study confirms the feasibility of this strategy in a real-world multicenter setting, including the use of alternative donors and predominantly reduced-intensity conditioning regimens, further supporting its applicability across a broad spectrum of patients.

This study has several limitations. Its retrospective design and the heterogeneity of conditioning regimens and GVHD prophylaxis across centers may have introduced bias and limited the generalizability of the results. In addition, the proportion of refractory patients was small, so our findings mainly reflect outcomes in those who achieved at least a partial response to AZA/VEN prior to HSCT. The relatively short follow-up also prevents firm conclusions on long-term survival and late complications. Although the pair-matched analysis provides supportive evidence, prospective (ideally randomized) studies are needed to confirm these observations and to better delineate the role of AZA/VEN as a bridge-to-transplant strategy in R/R AML.

Despite these caveats, our data suggest that AZA/VEN may represent an attractive option for patients with R/R AML, particularly older patients or those with significant comorbidities who are less likely to tolerate intensive chemotherapy. The rapid time to response further emphasizes the value of early assessment—after the first cycle and including MRD evaluation—to inform therapeutic decisions, whether to proceed directly to transplantation, continue additional cycles, or consider treatment intensification. Future randomized studies, ideally integrating novel VEN-based combinations, will be essential to validate this approach and optimize long-term outcomes in this high-risk population.

## Supplementary information

Supplementary Figures and Tables.

## Data Availability

The datasets generated and analyzed during the current study are available from the corresponding author on reasonable request, under regulatory conditions.

## References

[1] Döhner H, Wei AH, Appelbaum FR, Craddock C, DiNardo CD, Dombret H, et al. Diagnosis and management of AML in adults: 2022 recommendations from an international expert panel on behalf of the ELN. Blood. 2022;140:1345–77. 35797463 10.1182/blood.2022016867

[2] Thol F, Döhner H, Ganser A. How I treat refractory and relapsed acute myeloid leukemia. Blood. 2024;143:11–20. 37944143 10.1182/blood.2023022481

[3] Ganzel C, Sun Z, Cripe LD, Fernandez HF, Douer D, Rowe JM, et al. Very poor long-term survival in past and more recent studies for relapsed AML patients: the ECOG-ACRIN experience. Am J Hematol. 2018;93:1074–81. 29905379 10.1002/ajh.25162 PMC6699929

[4] DiNardo CD, Jonas BA, Pullarkat V, Thirman MJ, Garcia JS, Wei AH, et al. Azacitidine and venetoclax in previously untreated acute myeloid leukemia. N Engl J Med. 2020;383:617–29. 32786187 10.1056/NEJMoa2012971

[5] Aldoss I, Yang D, Aribi A, Ali H, Sandhu K, Al Malki MM, et al. Efficacy of the combination of venetoclax and hypomethylating agents in relapsed/refractory acute myeloid leukemia. Haematologica. 2018;103:e404–e407. 29545346 10.3324/haematol.2018.188094 PMC6119155

[6] Feld J, Tremblay D, Dougherty M, Czaplinska T, Sanchez G, Brady C, et al. Safety and efficacy: clinical experience of venetoclax in combination with hypomethylating agents in both newly diagnosed and relapsed/refractory advanced myeloid malignancies. Hemasphere. 2021; 5:e549. 10.1097/HS9.0000000000000549 PMC7951133 33718803

[7] Labrador J, Saiz-Rodríguez M, de Miguel D, de La Iglesia A, Rodríguez-Medina C, Vidriales MB, et al. Use of venetoclax in patients with relapsed or refractory acute myeloid leukemia: the PETHEMA registry experience. Cancers. 2022;14:1734. 35406512 10.3390/cancers14071734 PMC8997036

[8] Garciaz S, Hospital MA, Alary AS, Saillard C, Hicheri Y, Mohty B, et al. Azacitidine plus venetoclax for the treatment of relapsed and newly diagnosed acute myeloid leukemia patients. Cancers. 2022;14:2025. 35454930 10.3390/cancers14082025 PMC9028084

[9] Jamy O, Lin K, Worth S, Bachiashvili K, Rangaraju S, Vachhani P, et al. Hypomethylating agent/venetoclax versus intensive chemotherapy in adults with relapsed or refractory acute myeloid leukaemia. Br J Haematol. 2022;198:e35–e37. 35509246 10.1111/bjh.18229

[10] Angotzi F, Lessi F, Leoncin M, Filì C, Endri M, Lico A, et al. Efficacy and safety of venetoclax plus hypomethylating agents in relapsed/refractory acute myeloid leukemia: a multicenter real-life experience. Front Oncol. 2024;14:1370405. 10.3389/fonc.2024.1370405. 38680863 10.3389/fonc.2024.1370405 PMC11045980

[11] Petit C, Higue J, Acheaibi Z, Gilhodes J, Hospital MA, Devillier R, et al. Venetoclax-azacitidine versus azacitidine for the treatment of primary refractory or first relapsed acute myeloid leukemia. An IPC-DATAML-MSKCC retrospective study. Am J Hematol. 2025;100:906–8. 40088036 10.1002/ajh.27626

[12] Shahswar R, Gabdoulline R, Krueger K, Wichmann M, Götze KS, Braitsch K, et al. A novel prognostic risk model for patients with refractory/relapsed acute myeloid leukemia receiving venetoclax plus hypomethylating agents. Leukemia. 2025;39:614–22. 39779979 10.1038/s41375-024-02501-6 PMC11879869

[13] Pasvolsky O, Shimony S, Ram R, Shimoni A, Shargian L, Avni B, et al. Allogeneic hematopoietic cell transplantation for acute myeloid leukemia in first complete remission after 5-azacitidine and venetoclax: a multicenter retrospective study. Ann Hematol. 2022;101:379–87. 34628534 10.1007/s00277-021-04693-8

[14] Pollyea DA, Winters A, McMahon C, Schwartz M, Jordan CT, Rabinovitch R, et al. Venetoclax and azacitidine followed by allogeneic transplant results in excellent outcomes and may improve outcomes versus maintenance therapy among newly diagnosed AML patients older than 60. Bone Marrow Transpl. 2022;57:160–6. 10.1038/s41409-021-01476-7 34645926

[15] Winters AC, Bosma G, Abbott D, Minhajuddin M, Jordan C, Pollyea DA, et al. Outcomes are similar after allogeneic hematopoietic stem cell transplant for newly diagnosed acute myeloid leukemia patients who received venetoclax + azacitidine versus intensive chemotherapy. Transpl Cell Ther. 2022;28:694.e1–694.e9. 10.1016/j.jtct.2022.07.022 35902048

[16] Short NJ, Ong F, Ravandi F, Nogueras-González G, Kadia TM, Daver N, et al. Impact of type of induction therapy on outcomes in older adults with AML after allogeneic stem cell transplantation. Blood Adv. 2023;7:3573–81. 37104058 10.1182/bloodadvances.2022009632 PMC10368841

[17] Yang TT, Song XL, Zhao YM, Ye BD, Luo Y, Xiao HW, et al. Outcome after allogeneic hematopoietic stem cell transplantation following Venetoclax-based therapy among AML and MDS patients. Ann Hematol. 2022;101:2731–41. 36318288 10.1007/s00277-022-04983-9

[18] Bang SY, Park S, Kwag D, Lee JH, Min GJ, Park SS, et al. A successful bridge therapy combining hypomethylating agents with venetoclax for adult patients with newly diagnosed or relapsed/refractory acute myeloid leukemia. Cancers. 2023;15:1666. 36980551 10.3390/cancers15061666 PMC10046472

[19] Unglaub JM, Schlenk RF, Middeke JM, Krause SW, Kraus S, Einsele H, et al. Venetoclax-based salvage therapy as a bridge to transplant is feasible and effective in patients with relapsed/refractory AML. Blood Adv. 2025;9:375–85. 39293081 10.1182/bloodadvances.2024013086 PMC11787451

[20] Ye Y, Liu X, Dai H, Hu J, Yu G, Zhang Y, et al. Venetoclax plus azacitidine with or without homoharringtonine followed by allogeneic haematopoietic cell transplantation in patients with relapsed/refractory acute myeloid leukaemia: a multicentre cohort study. Br J Haematol. 2025;207:151–61. 40399765 10.1111/bjh.20147

[21] Döhner H, DiNardo CD, Appelbaum FR, Craddock C, Dombret H, Ebert BL, et al. Genetic risk classification for adults with AML receiving less-intensive therapies: the 2024 ELN recommendations. Blood. 2024;144:2169–73. 39133932 10.1182/blood.2024025409

[22] Bacigalupo A, Ballen K, Rizzo D, Giralt S, Lazarus H, Ho V, et al. Defining the intensity of conditioning regimens: working definitions. Biol Blood Marrow Transpl. 2009;15:1628–33. 10.1016/j.bbmt.2009.07.004 PMC2861656 19896087

[23] Schuurhuis GJ, Heuser M, Freeman S, Béné MC, Buccisano F, Cloos J, et al. Minimal/measurable residual disease in AML: a consensus document from the European LeukemiaNet MRD Working Party. Blood. 2018;131:1275–91. 29330221 10.1182/blood-2017-09-801498 PMC5865231

[24] Heuser M, Freeman SD, Ossenkoppele GJ, Buccisano F, Hourigan CS, Ngai LL, et al. 2021 Update on MRD in acute myeloid leukemia: a consensus document from the European LeukemiaNet MRD Working Party. Blood. 2021;138:2753–67. 34724563 10.1182/blood.2021013626 PMC8718623

[25] Plesa A, Dumontet C, Mattei E, Tagoug I, Hayette S, Sujobert P, et al. High frequency of CD34+CD38-/low immature leukemia cells is correlated with unfavorable prognosis in acute myeloid leukemia. World J Stem Cells. 2017;9:227–34. 29321824 10.4252/wjsc.v9.i12.227 PMC5746643

[26] Bleyzac N, Cuzzubbo D, Rénard C, Garnier N, Dubois V, Domenech C, et al. Improved outcome of children transplanted for high-risk leukemia by using a new strategy of cyclosporine-based GVHD prophylaxis. Bone Marrow Transpl. 2016;51:698–704. 10.1038/bmt.2015.350 26808568

[27] Przepiorka D, Weisdorf D, Martin P, Klingemann HG, Beatty P, Hows J, et al. 1994 consensus conference on acute GVHD grading. Bone Marrow Transpl. 1995;15:825–8. 7581076

[28] Shulman HM, Sullivan KM, Weiden PL, McDonald GB, Striker GE, Sale GE, et al. Chronic graft-versus-host syndrome in man. A long-term clinicopathologic study of 20 Seattle patients. Am J Med. 1980;69:204–17. 6996481 10.1016/0002-9343(80)90380-0

[29] Jagasia MH, Greinix HT, Arora M, Williams KM, Wolff D, Cowen EW, et al. National Institutes of Health Consensus Development Project on Criteria for Clinical Trials in Chronic Graft-versus-Host Disease: I. The 2014 Diagnosis and Staging Working Group report. Biol Blood Marrow Transpl. 2015;21:389–401.e1. 10.1016/j.bbmt.2014.12.001 PMC4329079 25529383

[30] Sorror ML, Maris MB, Storb R, Baron F, Sandmaier BM, Maloney DG, et al. Hematopoietic cell transplantation (HCT)-specific comorbidity index: a new tool for risk assessment before allogeneic HCT. Blood. 2005;106:2912–9. 15994282 10.1182/blood-2005-05-2004 PMC1895304

[31] Dumas PY, Bertoli S, Santana C, Heiblig M, Leguay T, Tavitian S, et al. Real-world multicenter comparison of venetoclax and azacitidine versus intermediate or high dose cytarabine-based salvage for patients with AML in first relapse after front-line intensive chemotherapy. Dataml-IPC-Auraml Consortium Study. Blood. 2024;144:4275.

[32] Hahn T, McCarthy PL Jr, Hassebroek A, Bredeson C, Gajewski JL, Hale GA, et al. Significant improvement in survival after allogeneic hematopoietic cell transplantation during a period of significantly increased use, older recipient age, and use of unrelated donors. J Clin Oncol. 2013;31:2437–49. 23715573 10.1200/JCO.2012.46.6193 PMC3691359

[33] Higué J, Orvain C, Dumas PY, Peterlin P, Hospital MA, Barrière S, et al. Venetoclax and azacitidine for molecular relapse after intensive chemotherapy in NPM1 or CBF AML: a FILO study. Blood Cancer J. 2025;15:141. 40841520 10.1038/s41408-025-01344-3 PMC12371059

[34] Walter RB, Buckley SA, Pagel JM, Wood BL, Storer BE, Sandmaier BM, et al. Significance of minimal residual disease before myeloablative allogeneic hematopoietic cell transplantation for AML in first and second complete remission. Blood. 2013;122:1813–21. 23847197 10.1182/blood-2013-06-506725 PMC3765060

[35] Gilleece MH, Labopin M, Yakoub-Agha I, Volin L, Socié G, Ljungman P, et al. Measurable residual disease, conditioning regimen intensity, and age predict outcome of allogeneic hematopoietic cell transplantation for acute myeloid leukemia in first remission: a registry analysis of 2292 patients by the Acute Leukemia Working Party European Society of Blood and Marrow Transplantation. Am J Hematol. 2018;93:1142–52. 29981272 10.1002/ajh.25211

[36] Gilleece MH, Shimoni A, Labopin M, Robinson S, Beelen D, Socié G, et al. Measurable residual disease status and outcome of transplant in acute myeloid leukemia in second complete remission: a study by the acute leukemia working party of the EBMT. Blood Cancer J. 2021;11:88. 33980810 10.1038/s41408-021-00479-3 PMC8116335

[37] Jentzsch M, Bischof L, Backhaus D, Brauer D, Schulz J, Franke GN, et al. Impact of MRD status in patients with AML undergoing allogeneic stem cell transplantation in the first vs the second remission. Blood Adv. 2022;6:4570–80. 35605254 10.1182/bloodadvances.2022007168 PMC9636320

