# Supplementary material for: Allogeneic hematopoietic stem cell transplantation after azacitidine and venetoclax salvage in relapsed/refractory AML: a multicenter real-world study by the French AURAML group
Source: Bone Marrow Transplant. 2026 Mar 24;61(5):551–8. doi: 10.1038/s41409-026-02834-z (PMC13152806; doi:10.1038/s41409-026-02834-z)
Supplement: Supplementary file 1 — Supplementary Figures and Tables. [file 41409_2026_2834_MOESM1_ESM.pdf]

Supplemental figure 1

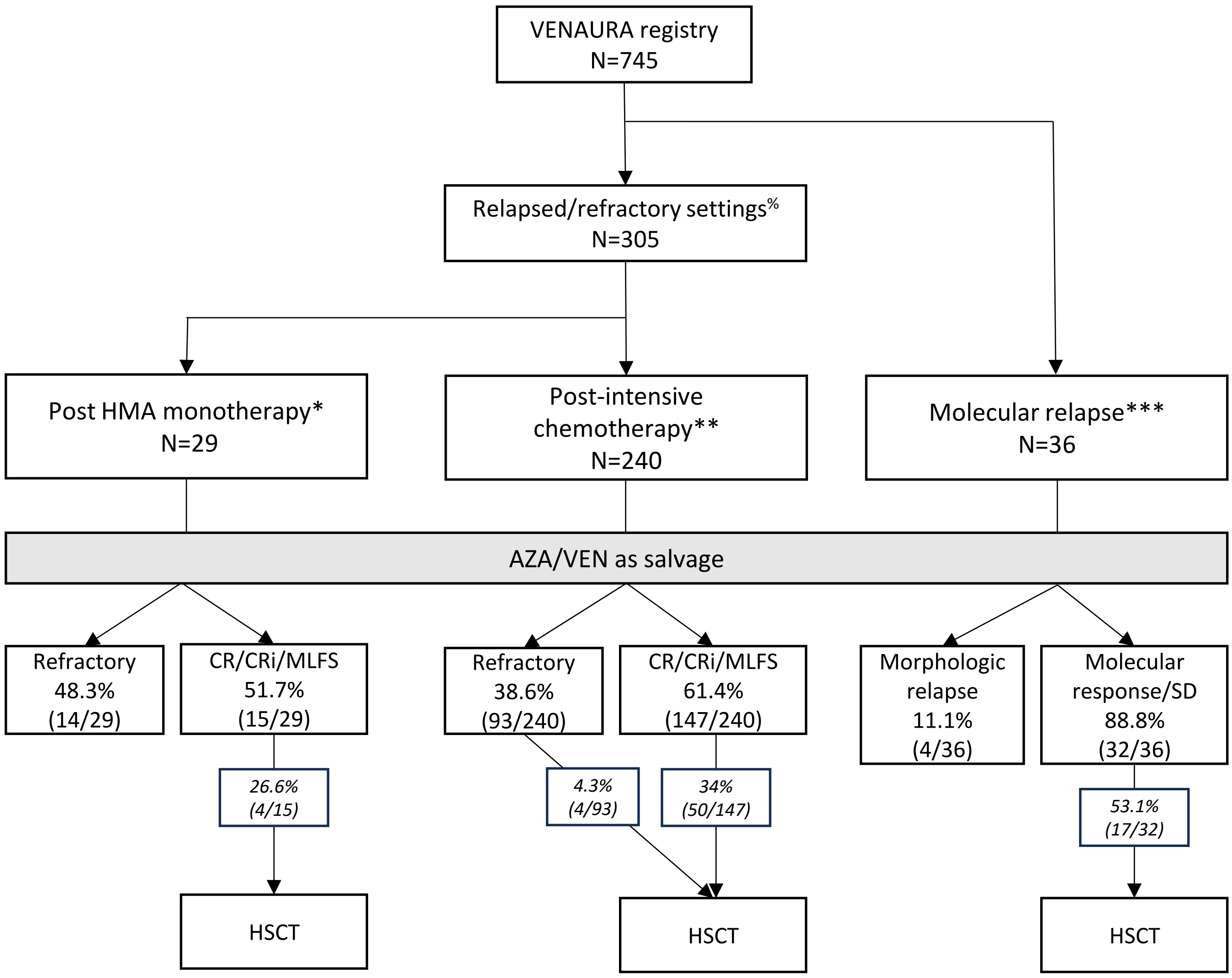

Supplemental figure 1. Patients flow chart.

Legend: AZA, azacitidine ; CR, complete response ; CRi, complete response with incomplete hematologic recovery ; HMA, hypomethylating agent ; HSCT, allogeneic hematopoietic stem cell transplantation ; MLFS, morphologic leukemia-free state ; SD, stable disease ; VEN, venetoclax

* Patients received HMA as a single agent for high-risk myelodysplastic syndrome that secondarily progressed to acute myeloid leukemia

** Among patients treated with AZA/VEN as a salvage in R/R settings post-intensive chemotherapy, 63 (26.3%) were in relapse after a first HSCT.

*** All patients in molecular relapse received intensive chemotherapy as a first line +/- subsequent HSCT

% Only patients evaluated at least 1 time for morphological response during the 6 first AZA/VEN cycles were included in this analysis

Supplemental figure 2

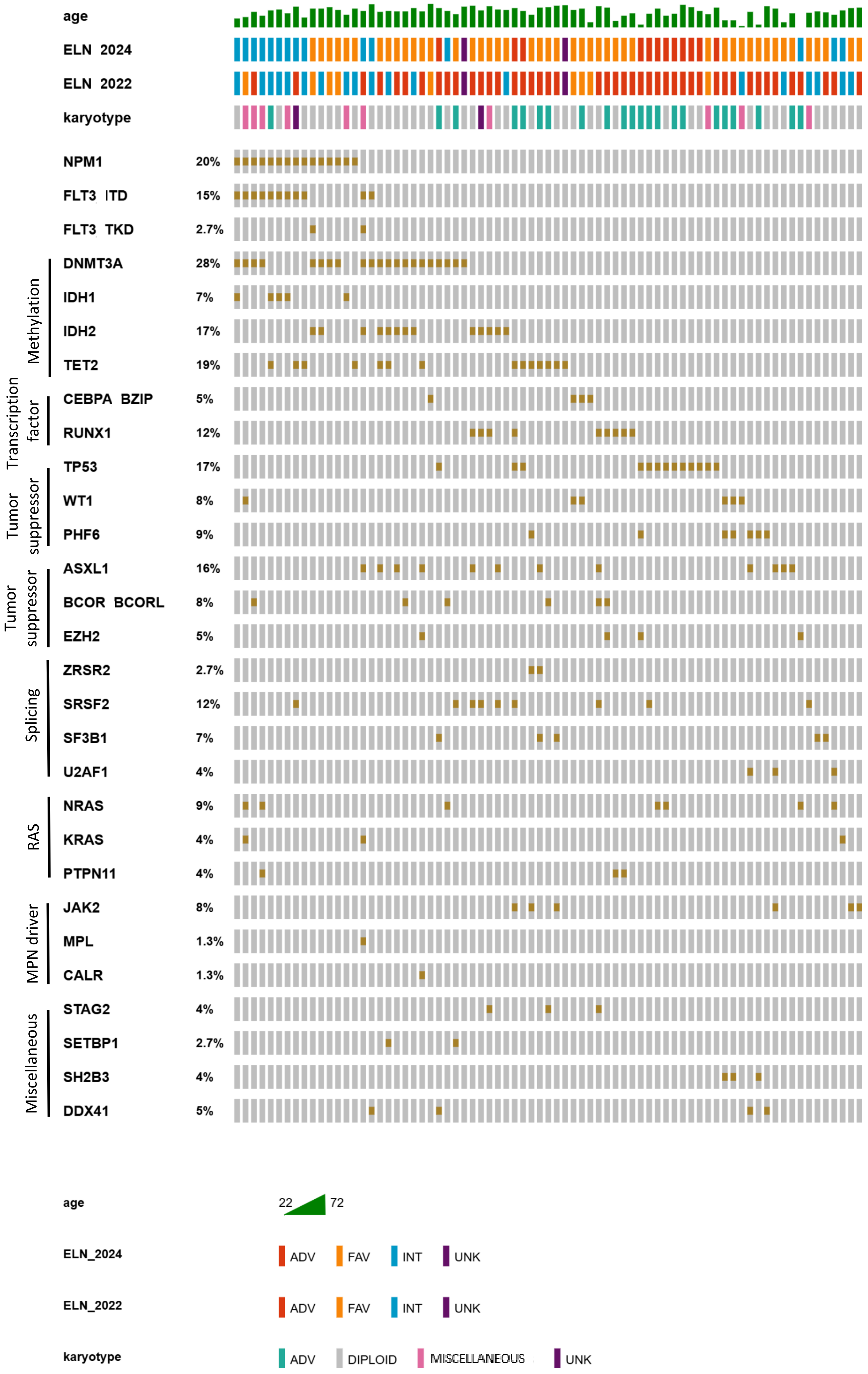

Supplemental figure 2. Oncoplot of patients molecular characteristics.
Legend: ADV, adverse ; ELN, European LeukemiaNet ; FAV, favorable ; INT, intermediate ; MPN, myeloproliferative neoplasm ; UNK, unknown

Supplemental figure 3

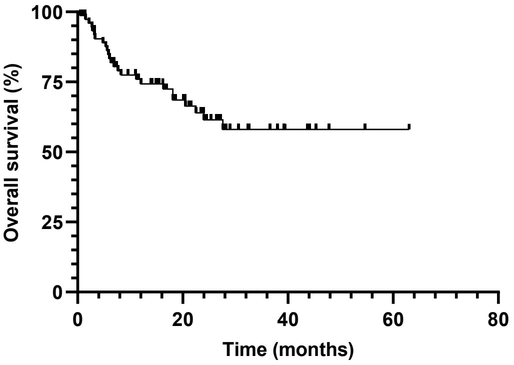

Supplemental figure 3. Kaplan-Meier estimate of post-HSCT OS in AZA/VEN treated patients.

Supplemental figure 4

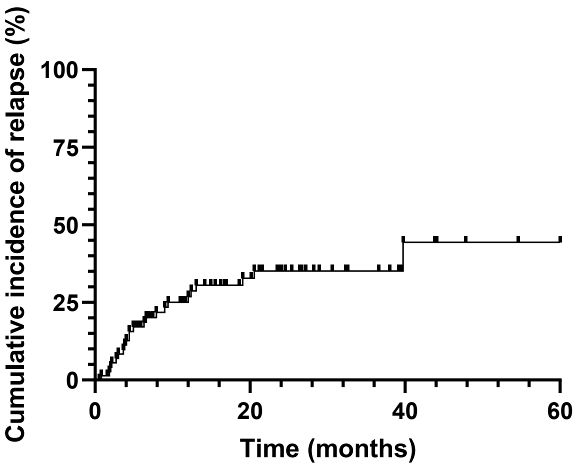

Supplemental figure 4. Kaplan-Meier estimate of post-HSCT CIR in AZA/VEN treated patients.

Supplemental figure 5

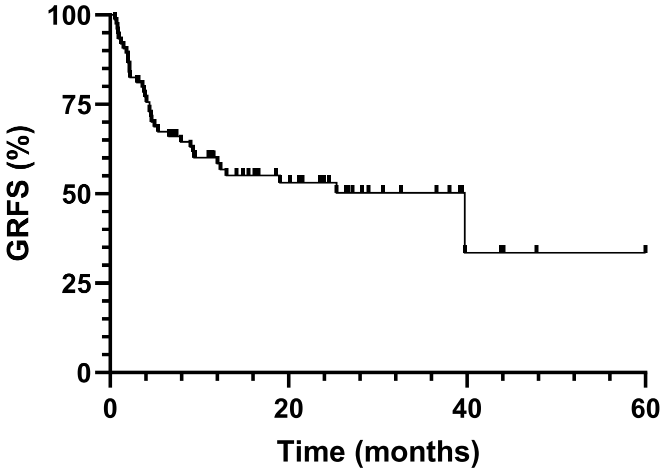

Supplemental figure 5. Kaplan-Meier estimate of post-HSCT GRFS in AZA/VEN treated patients.

Supplemental table 1 : Detailed conditioning regimen

Conditioning regimen	N=75
FB2	21/75
FB3	2/75
FB4	6/75
TBF	6/75
Reduced intensity TBF	15/75
Baltimore	5/75
TTF	1/75
FluMel	1/75
TreoFlu	3/75
FluTBI (+/- Cy)	3/75
FLAMSA-BuCy2	8/75
FLAMSA-Bu4	3/75
TEC-RIC	1/75

Supplemental table 1. Detailed conditioning regimens of AZA/VEN treated patients.
Legend : FB2, fludarabine and 2 days of busulfan ; FB3, fludarabine and 3 days of busulfan ; fludarabine and 4 days of busulfan ; TBF, thiotepa, busulfan and fludarabine ; Reduced intensity TBF, TBF with reduced dose busulfan ; Baltimore, fludarabine, total body irradiation and post-transplant cyclophosphamide ; TTF, thiotepa, treosulfan and fludarabine ; FluMel, fludarabine and melphalan ; TreoFlu, treosulfan and fludarabine ; FluTBI, fludarabine and total body irradiation ; Cy, cyclophosphamide ; FLAMSA-BuCy2, fludarabine, cytarabine, amsacrine with busulfan and 2 days of cyclophosphamide ; FLAMSA-Bu4, fludarabine, cytarabine and amsacrine with 4 days of busulfan ; TEC-RIC, thiotepa, etoposide and cyclophosphamide with fludarabine and busulfan.

Supplemental table 2 : OS univariate analysis

Variables	Comparison	HR	95%CI low	95%CI high	p-value
Sex	Male vs female	1.63	0.72	3.7	0.24
Age	Continuous	1.03	0.98	1.07	0.22
AML Type	MR vs *de novo*	3.47	1.38	8.75	0.008
	sAML vs *de novo*	1.91	0.68	5.38	0.22
Karyotype	Miscellaneous vs diploid	0.32	0.04	2.49	0.28
	Adverse vs diploid	2.52	1.11	5.75	0.028
ELN 2024 risk classification	Favorable vs adverse	0.41	0.16	1.03	0.057
	Intermediate vs adverse	0.33	0.1	1.12	0.076
ELN 2022 risk classification	Favorable vs adverse	0.53	0.12	2.3	0.40
	Intermediate vs adverse	0.55	0.2	1.5	0.24
AZA/VEN cycle 1 response	No response vs CR/CRi/MLFS	5.41	1.97	14.87	0.001
AML status at AZA/VEN onset	Relapse vs refractory	0.82	0.37	1.81	0.62
Number of lines prior to AZA/VEN	One vs more	1.76	0.6	5.13	0.30
Number of AZA/VEN cycles pre-HSCT	Continuous	1.08	0.77	1.5	0.66
Pre-HSCT cytologic response	CRi/MLFS vs CR	1.65	0.6	4.52	0.33
	Refractory vs CR	3.98	1.15	13.72	0.029
Pre-HSCT MRD	MRD^{pos} vs MRD^{neg}	1.48	0.64	3.39	0.36
Time from diagnosis to HSCT (months)	Continuous	1	0.97	1.03	0.92
Time from CR to HSCT (months)	Continuous	1	1	1	0.67
Donor type	HAPLO vs MSD	1.26	0.31	5.05	0.75
	MMUD vs MSD	4.99	0.96	26	0.06
	MUD vs MSD	1.32	0.38	4.64	0.66
HSCT conditioning regimen	RIC vs MAC	0.81	0.24	2.76	0.73
	SEQ vs MAC	0.79	0.19	3.33	0.75
HCT-CI score	1 vs 0	0.52	0.07	4.02	0.53
	2 vs 0	1.09	0.38	3.13	0.88
	≥3 vs 0	1.6	0.51	5.04	0.42

Supplemental table 2. Univariate analysis of post-HSCT OS in AZA/VEN treated patients.
Legend : 95%CI, 95% confidence interval ; AML, acute myeloid leukemia ; AZA/VEN, azacitidine and venetoclax ; CR, complete response ; CRi, Complete response with incomplete hematological recovery ; ELN, European LeukemiaNet ; HAPLO, haplo-identical ; HCT-CI, hematopoietic cell transplantation-specific comorbidity index ; HR, hazards ratio ; HSCT, allogeneic hematopoietic stem cell transplantation ; MAC, myeloablative conditionning ; MLFS, morphologic leukemia-free state ; MMUD, mismatched unrelated donor ; MR, myelodysplasia-related ; MRD, minimal residual disease ; MSD, matched sibling donor ; MUD, matched unrelated donor ; RIC, reduced intensity conditionning ; sAML, secondary acute myeloid leukemia; SEQ, sequential conditioning regimen.

Supplemental table 3 : CIR univariate analysis

Variables	Comparison	HR	95%CI low	95%CI high	p-value
Sex	Male vs female	1.03	0.99	1.08	0.12
Age	Continuous	2.91	1.15	7.37	0.024
AML Type	MR vs *de novo*	1.82	0.6	5.49	0.29
	sAML *vs de novo*	1.93	0.69	5.38	0.21
Karyotype	Miscellaneous vs diploid	0.43	0.05	3.36	0.42
	Adverse vs diploid	4.27	1.84	9.93	0.0007
ELN 2024 risk classification	Favorable vs adverse	0.22	0.09	0.55	0.001
	Intermediate vs adverse	0.13	0.03	0.51	0.003
ELN 2022 risk classification	Favorable vs adverse	0.85	0.25	2.93	0.80
	Intermediate vs adverse	0.55	0.2	1.52	0.25
AZA/VEN cycle 1 response	No response vs CR/CRi/MLFS	2.6	1.1	6.6	0.033
AML status at AZA/VEN onset	Relapse vs refractory	0.74	0.33	1.68	0.478
Number of lines prior to AZA/VEN	One vs more	0.92	0.36	2.33	0.86
Number of AZA/VEN cycles pre-HSCT	Continuous	0.76	0.5	1.15	0.19
Pre-HSCT cytologic response	CRi/MLFS vs CR	1.88	0.68	5.21	0.23
	Refractory vs CR	4.3	1.18	15.6	0.027
PreHSCT MRD	MRD^{pos} vs MRD^{neg}	2.55	1.14	5.7	0.023
Time from diagnosis to HSCT (months)	Continuous	0.99	0.95	1.02	0.47
Time from CR to HSCT (months)	Continuous	1	1	1	0.44
Donor type	HAPLO vs MSD	1.2	0.3	4.8	0.80
	MMUD vs MSD	4.45	0.86	22.98	0.07
	MUD vs MSD	1.35	0.38	4.74	0.64
HSCT conditioning regimen	RIC vs MAC	3.3	0.44	24.72	0.24
	SEQ vs MAC	2.15	0.24	19.34	0.49
HCT-CI score	1 vs 0	1.06	0.22	5.07	0.94
	2 vs 0	1.19	0.39	3.57	0.76
	≥3 vs 0	1.55	0.42	5.78	0.51

Supplemental table 3. Univariate analysis of post-HSCT CIR in AZA/VEN treated patients.
Legend : 95%CI, 95% confidence interval ; AML, acute myeloid leukemia ; AZA/VEN, azacitidine and venetoclax ; CR, complete response ; CRi, Complete response with incomplete hematological recovery ; ELN, European LeukemiaNet ; HAPLO, haplo-identical ; HCT-CI, hematopoietic cell transplantation-specific comorbidity index ; HR, hazards ratio ; HSCT, allogeneic hematopoietic stem cell transplantation ; MAC, myeloablative conditionning ; MLFS, morphologic leukemia-free state ; MMUD, mismatched unrelated donor ; MR, myelodysplasia-related ; MRD, minimal residual disease ; MSD, matched sibling donor ; MUD, matched unrelated donor ; RIC, reduced intensity conditionning ; sAML, secondary acute myeloid leukemia; SEQ, sequential conditioning regimen.

Supplemental table 4 : GRFS univariate analysis

Variables	Comparison	HR	95%CI low	95%CI high	p-value
Sex	Male vs Female	1.68	0.82	3.41	0.15
Age	Continuous	1.03	0.99	1.06	0.16
AML Type	MR vs *de novo*	2.98	1.3	6.8	0.009
	sAML vs *de novo*	1.21	0.45	3.24	0.71
Karyotype	Miscellaneous vs diploid	0.22	0.03	1.69	0.15
	Adverse vs diploid	2.51	1.23	5.12	0.011
ELN 2024 risk classification	Favorable vs adverse	0.32	0.14	0.7	0.004
	Intermediate vs Adverse	0.16	0.05	0.52	0.002
ELN 2022 risk classification	Favorable vs adverse	0.5	0.15	1.65	0.25
	Intermediate vs Adverse	0.46	0.19	1.1	0.08
AML status at AZA/VEN onset	Relapse vs Refractory	0.63	0.31	1.27	0.19
AZA/VEN cycle 1 response	No response *vs* CR/CRi/MLFS	5.09	1.93	13.4	0.001
Number of lines prior to AZA/VEN	One vs more	0.7	0.3	1.63	0.41
Number of AZA/VEN cycles pre-HSCT	Continuous	1.01	0.75	1.37	0.94
pre-HSCT cytologic response	CRi/MLFS vs CR	1.76	0.74	4.15	0.2
	Refractory vs CR	2.56	0.74	8.88	0.14
Pre-HSCT MRD	MRD^{pos} vs MRD^{neg}	2.12	1.06	4.24	0.03
Time from diagnosis to HSCT (months)	Continuous	0.99	0.96	1.02	0.44
Time from CR to HSCT (months)	Continuous	1	1	1	0.88
Donor type	MMUD vs HAPLO	1.26	0.42	3.83	0.68
	MSD vs HAPLO	2.44	0.57	10.51	0.23
	MUD vs HAPLO	1.24	0.46	3.33	0.67
HSCT conditioning regimen	RIC vs MAC	1.16	0.35	3.87	0.81
	SEQ vs MAC	1.36	0.35	5.28	0.65
GVHD prophylaxis	CSA+MMF vs CSA	0.98	0.45	2.15	0.96
	CSA+MTX vs CSA	1.48	0.5	4.38	0.48
HCT-CI score	1 vs 0	0.75	0.16	3.41	0.71
	2 vs 0	1.71	0.72	4.08	0.22
	≥3 vs 0	1.64	0.57	4.67	0.36

Supplemental table 4. Univariate analysis of post-HSCT GRFS in AZA/VEN treated patients.
Legend : 95%CI, 95% confidence interval ; AML, acute myeloid leukemia ; AZA/VEN, azacitidine and venetoclax ; CR, complete response ; CRi, Complete response with incomplete hematological recovery ; CSA, cyclosporine ; ELN, European LeukemiaNet ; GVHD, graft-versus-host disease ; HAPLO, haplo-identical ; HCT-CI, hematopoietic cell transplantation-specific comorbidity index ; HR, hazards ratio ; HSCT, allogeneic hematopoietic stem cell transplantation ; MAC, myeloablative conditionning ; MLFS, morphologic leukemia-free state ; MMF, mycophenolate mofetil ; MMUD, mismatched unrelated donor ; MR, myelodysplasia-related ; MRD, minimal residual disease ; MSD, matched sibling donor ; MTX, methotrexate ; MUD, matched unrelated donor ; RIC, reduced intensity conditionning ; sAML, secondary acute myeloid leukemia; SEQ, sequential conditioning regimen.

Supplemental table 5 : NRM univariate analysis

Variables	Comparison	HR	95%CI low	95%CI high	p-value
Sex	Male vs Female	0.36	0.07	1.85	0.221
Age	Continuous	1.03	0.95	1.11	0.489
AML Type	MR vs *de novo*	9.08	1.51	54.54	0.016
	sAML *vs de novo*	4.71	0.66	33.64	0.122
Karyotype	Miscellaneous vs diploid	0	0	Inf	0.998
	Adverse vs diploid	0.44	0.05	3.78	0.454
ELN 2024 risk classification	Favorable vs adverse	0	0	Inf	0.998
	Intermediate vs adverse	0	0	Inf	0.998
ELN 2022 risk classification	Favorable vs adverse	0	0	Inf	1.00
	Intermediate vs adverse	0.45	0.05	3.82	0.46
AML status at AZA/VEN onset	Relapse vs Refractory	1.85	0.21	16.15	0.577
AZA/VEN cycle 1 response	No response *vs* CR/CRi/MLFS	1.33	0.3	5.95	0.708
Number of lines prior to AZA/VEN	One vs more	0	0	Inf	0.999
Number of AZA/VEN cycles pre-HSCT	Continuous	1.43	0.89	2.28	0.138
pre-HSCT cytologic response	CRi/MLFS vs CR	1.81	0.35	9.39	0.480
	Refractory vs CR	0	0	Inf	0.999
Pre-HSCT MRD	MRD^{pos} *vs* MRD^{neg}	0.47	0.06	4.07	0.497
Time from diagnosis to HSCT (months)	Continuous	1.01	0.96	1.05	0.782
Time from CR to HSCT (months)	Continuous	0.96	0.65	1.42	0.836
Donor type	HAPLO vs MSD	0	0	Inf	1.00
	MMUD vs MSD	1.65	0.14	19.03	0.69
	MUD vs MSD	0.59	0.11	3.21	0.54
HSCT conditioning regimen	RIC vs MAC	0.31	0.06	1.67	0.172
	SEQ vs MAC	0.3	0.03	3.3	0.325
GVHD prophylaxis	CSA+MMF vs CSA	0	0	Inf	0.999
	CSA+MTX vs CSA	2.01	0.44	9.13	0.363
HCT-CI score	1 vs 0	2.84	0.26	31.3	0.395
	2 vs 0	1.17	0.11	12.92	0.898
	≥3 vs 0	5.75	0.96	34.53	0.056

Supplemental table 5. Univariate analysis of post-HSCT NRM in AZA/VEN treated patients.
Legend : 95%CI, 95% confidence interval ; AML, acute myeloid leukemia ; AZA/VEN, azacitidine and venetoclax ; CR, complete response ; CRi, Complete response with incomplete hematological recovery ; CSA, cyclosporine ; ELN, European LeukemiaNet ; GVHD, graft-versus-host disease ; HAPLO, haplo-identical ; HCT-CI, hematopoietic cell transplantation-specific comorbidity index ; HR, hazards ratio ; HSCT, allogeneic hematopoietic stem cell transplantation ; MAC, myeloablative conditionning ; MLFS, morphologic leukemia-free state ; MMF, mycophenolate mofetil ; MMUD, mismatched unrelated donor ; MR, myelodysplasia-related ; MRD, minimal residual disease ; MSD, matched sibling donor ; MTX, methotrexate ; MUD, matched unrelated donor ; RIC, reduced intensity conditionning ; sAML, secondary acute myeloid leukemia; SEQ, sequential conditioning regimen.

Supplemental table 6 : baseline characteristics of pair-matched cohorts

Patients characteristics	AZA/VEN pre-HSCT N=75	IC salvage pre-HSCT N=75	P-value
**Age,** years, median (range)	58,2 (18-73)	58 (29.6-68.9)	0.91
**Female sex**, n (%)	36/75 (48)	38/75 (50.6)	0.94
**Adverse cytogenetics**, n (%)	22/75 (29.3)	16/75 (21.3)	0.26
**Intensive chemotherapy lines prior to AZA/VEN**, n (%)			
• 1	58/75 (77.3)	65/75 (86.6)	0.13
• 2	17/75 (22.7)	10/75 (13.4)	
**IC salvage type**, n (%)			
• IDAC-based	-	10/75 (13.3)	-
• Anthracyclines-based		46/75 (57.3)	
• GO-based		22/75 (29.4)	
**Pre-HSCT cytologic response**, n (%)			
• CR	55/75 (73.3)	60/75 (80)	0.25
• CRi/MLFS	16/75 (21.3)	10/75 (13.3)	
• Refractory	4/75 (5.4)	5/75 (6,7)	
**MFC pre-HSCT MRD**, n (%)			
• Negative	47/71 (66.2)	35/67 (52.2)	0.07
• Positive	24/71 (33.8)	24/67 (35.8)	
• Not performed	5/71 (7)	10/67 (14.9)	
**Donor type,** n (%)			
• MSD	12/75 (16.2)	26/75 (34.7)	0.055
• MUD	38/75 (50.6)	35/75 (46.7)	
• MMUD	5/75 (6.6)	4/75 (5.3)	
• HAPLO	20/75 (26.6)	10/75 (13.3)	
**HCT-CI**, n (%)			
• 0 - 1	41/75 (54.6)	40/75 (53.4)	0.07
• 2	16/75 (21.3)	8/75 (10.6)	
• ≥ 3	11/75 (14.7)	10/75 (13.3)	
• Missing	7/75 (9.4)	17/75 (22.7)	
**Conditioning regimens**, n (%)			
• RIC	53/75 (70.7)	41/75 (54.7)	0.07
• MAC	9/75 (12)	9/75 (12)	
• SEQ	13/75 (17.3)	25/75 (33.3)	
**Post transplantation GVHD prophylaxis**, n (%)			
• CSA	21/75 (28)	10/75 (13.3)	0.075
• CSA+MMF	42/75 (56)	53/75 (70.7)	
• CSA+MTX	12/75 (16)	12/75 (16)	

Supplemental table 6. Baseline characteristics of pair-matched AZA/VEN and IC treated patients.
Legend : 95%CI, 95% confidence interval ; AML, acute myeloid leukemia ; AZA/VEN, azacitidine and venetoclax ; CR, complete response ; CRi, Complete response with incomplete hematological recovery ; CSA, cyclosporine ; GO, gemtuzumab ozogamicin ; GVHD, graft-versus-host disease ; HAPLO, haplo-identical ; HCT-CI, hematopoietic cell transplantation-specific comorbidity index ; HSCT, allogeneic hematopoietic stem cell transplantation ; IC, intensive chemotherapy ; IDAC, intermediate dose cytarabine ; MAC, myeloablative conditionning ; MFC, multiparameter flow cytometry ; MLFS, morphologic leukemia-free state ; MMF, mycophenolate mofetil ; MMUD, mismatched unrelated donor ; MRD, minimal residual disease ; MSD, matched sibling donor ; MTX, methotrexate ; MUD, matched unrelated donor ; RIC, reduced intensity conditionning ; SEQ, sequential conditioning regimen.
